# Diagnostic relevance of urinary steroid profiles on ovarian granulosa cell tumors: two case reports

**DOI:** 10.1186/s13256-017-1324-1

**Published:** 2017-06-22

**Authors:** Anita Bufa, Nelli Farkas, Zsolt Preisz, Viktória Poór, Csilla Páger, Sándor Szukits, Bálint Farkas, Péter Miklós Gőcze

**Affiliations:** 1 Institute of Bioanalysis, Faculty of Medicine, University of Pécs, Szigeti street 12, Pécs, 7624 Hungary; 2 Department of Radiology, University of Pécs Clinical Centre, Ifjúság street 13, Pécs, 7624 Hungary; 3 Department of Obstetrics and Gynaecology, University of Pécs Clinical Centre, Édesanyák street 17, Pécs, 7624 Hungary; 4 MTA-PTE Human Reproduction Scientific Research Group, Hungarian Academy of Sciences (MTA), Pécs, Hungary

**Keywords:** Ovarian granulosa cell tumor, Urinary steroid profiles, Diagnostics, Gas chromatography-mass spectrometry

## Abstract

**Background:**

Granulosa cell tumor of the ovary is the most frequent sex cord stromal tumor and represents 2 to 5% of all primary ovarian cancers. Ovarian granulosa cell tumor is a malignant tumor with slow progression and in some cases this tumor is hormonally active. The recurrence of granulosa cell tumor often happens after 5 years.

**Case presentation:**

We describe two cases of postmenopausal women with adult-type granulosa cell tumors of the ovary. Patient 1 is a 49-year-old European woman with a recurrent tumor; patient 2 is a 55-year-old European woman without recurrence of tumor. Urinary steroid profiles of patient 1 were monitored during a 5-year period starting from before an operation (13 samples). In patient 2, the urinary steroid profiles were monitored during a 3-year period starting from after an operation (six samples). The 24-hour urinary samples were examined and the urinary concentration of 20 androgen, progesterone, and corticoid metabolites was quantitatively determined by gas chromatography-mass spectrometry with selected ion-monitoring mode.

**Conclusions:**

Based on these cases a correlation could be observed between increased levels of the urinary steroids and the recurrence of ovarian granulosa cell tumor; therefore, we concluded that a urinary steroid profile could be a more effective method to follow-up such patients compared to the traditional serum hormones determinations supplemented with conventional tumor markers.

## Background

Granulosa cell tumor (GCT) of the ovary is a malignant tumor originating from the sex-cord stromal cells of the ovary and represents approximately 5% of all primary ovarian cancers [1–3]. Approximately 4% occur before puberty (juvenile GCT) and the majority of the cases are the adult type of GCT (occur in people of reproductive age and postmenopausal age) [4]. In some cases these tumors are hormonally active, they often express steroid hormone receptors [3, 5]. The natural history of GCT is generally long with slow progression, and recurrence often happens after 5 years of follow-up [6]. We report two cases of adult GCT of the ovary and describe the changes experienced in urinary steroid profiles which could help in following-up the presence, progression, and recurrence of this tumor.

## Case presentation

### Patient 1

**Fig. 1:**
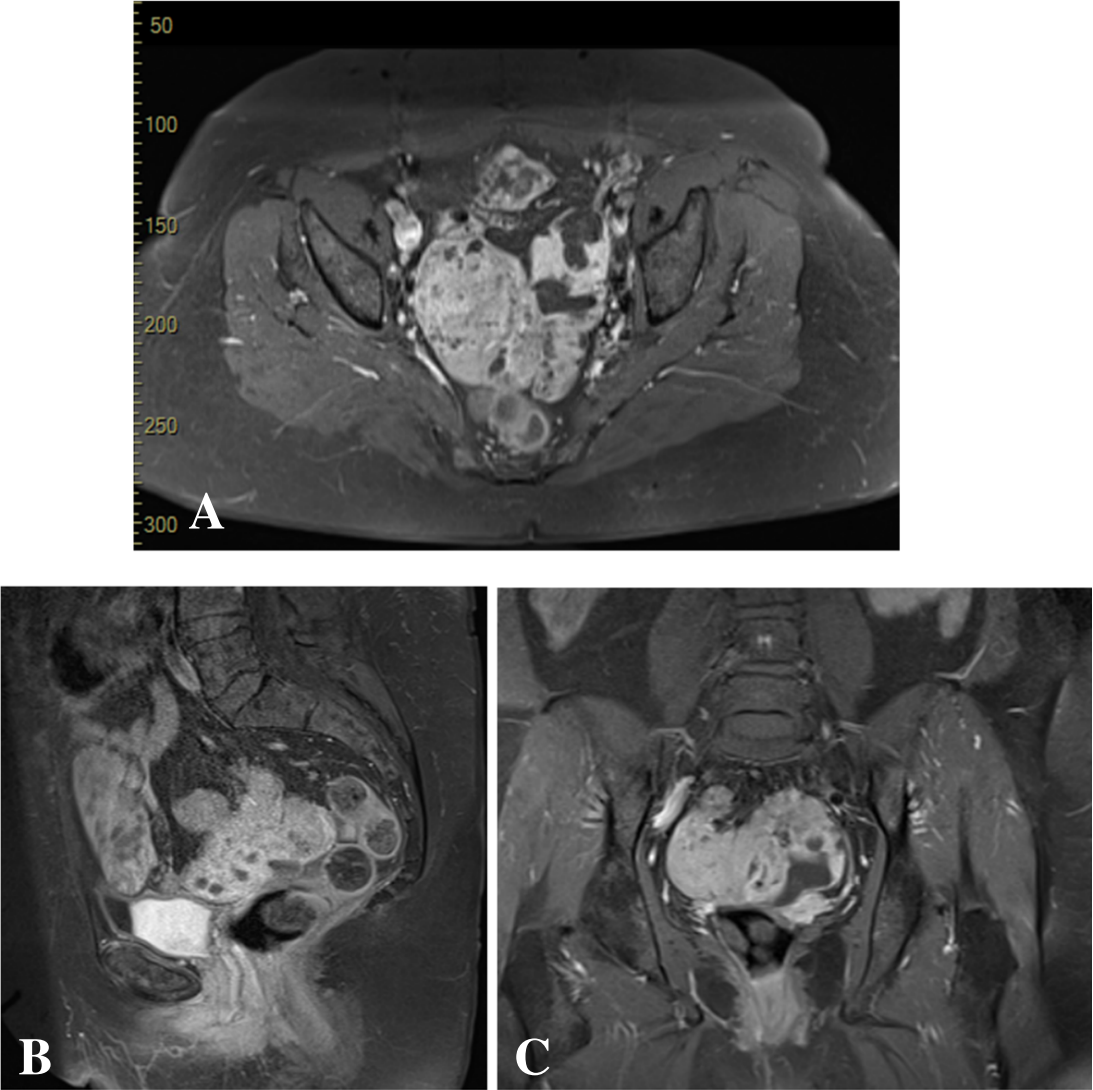
Patient 1 – magnetic resonance images of the recurrence of ovarian granulosa cell tumor. **a** Axial, **b** sagittal, and **c** coronal T2-weighted images showing a local tumor recurrence (87×108×70 mm), diffuse peritoneal metastasis (68×35 mm) with ascites from the pelvis, a parailiacal pathological lymph node (15×10 mm), and an inguinal pathological lymph node (62×32 mm) from the left side of the pelvis

**Fig. 2:**
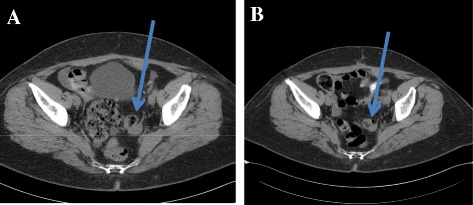
Patient 1 Computer tomography images of recurrent ovarian granulosa cell tumour. **a** The progression of tumour (local tumour recurrence (blue arrow), diffuse peritoneal carcinosis with ascites and inguinal pathological lymph node), and **b** the regression of local recurrence tumour (blue arrow)

**Fig. 3:**
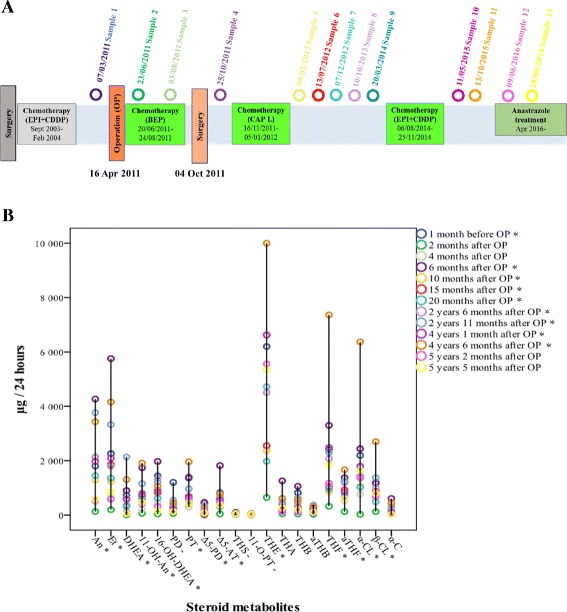
Patient 1 **a** The interventions and treatments of patient and the collection of urine samples. **b** The levels of the urinary steroid metabolites at different time points. * represents a steroid level that is higher than the same age and same sex reference value. **-** represents a steroid level that is lower than the reference value in all samples

A 49-year-old European, postmenopausal woman who was diagnosed with stage T1a ovarian GCT had recurrence 7 years after primary surgery and five cycles of chemotherapy: epirubicin + cisplatin. On admission, the results of her neurological examination were normal. A physical examination revealed two cystic masses above her vagina on the left and slightly to the right that varied from 6 to 10 cm in diameter. The results of the rest of her physical examination were normal. She had given birth to three children. In January 2011, magnetic resonance imaging (MRI) depicted a large pelvic mass with inhomogeneous signal intensity (87×108×70 mm), a diffuse peritoneal metastasis (68×35 mm) with ascites, a parailiacal pathological lymph node (15×10 mm), and an inguinal pathological lymph node (62×32 mm) from the left side of her pelvis (Fig. 1). A laboratory examination did not reveal elevated levels of serum tumor markers and hormones: carcinoembryonic antigen (CEA), 1.6 ng/ml; carbohydrate antigen-125 (CA-125), 18.45 U/ml; carbohydrate antigen-15-3 (CA-15-3), 20 U/ml; carbohydrate antigen-19-9 (CA-19-9), 0.6 U/ml; alpha-fetoprotein (AFP), 2.3 μg/l; follicle-stimulating hormone (FSH), 23.1 U/l; luteinizing hormone (LH), 38.9 U/l; progesterone (P), 2 nmol/l; 17β-estradiol (E_2_), 64 pmol/l; testosterone (T), 0.92 nmol/l; and androstenediol (A), 7.74 nmol/l. She underwent abdominal hysterectomy with bilateral salpingo-oophorectomy and omentectomy 3 months later. A histologic examination revealed recurrence of GCT. Immunohistochemical staining gave positive results for alpha-inhibin. After the operation (OP), she was given three cycles of chemotherapy: bleomycin, etoposide, and cisplatin (BEP). Two months later she underwent metastasectomy through laparotomy and she was given three cycles of third line chemotherapy: cyclophosphamide and doxorubicin (CAP I). Despite surgeries and chemotherapies, in August 2014 a computed tomography (CT) scan showed significant progression of the recurrent GCT, local tumor recurrence, the presence of diffuse peritoneal carcinosis, ascites, and inguinal pathological lymph node (Fig. 2). A laboratory examination did not reveal elevated levels of serum tumor markers: CEA, 1.3 ng/ml; CA-125, 13 U/ml. Then she was given three cycles of re-induction of epirubicin + cisplatin chemotherapy. Three months later in February 2015, CT demonstrated 25% regression of the tumor (Fig. 2). Then, she refused further parenteral chemotherapy. From April 2016 she received oral anastrozole (Arimidex®) therapy. During the treatments, before and after the OP, 13 24-hour urinary samples were collected at different time points. Her urine samples were stored at −20 ^○^C until analysis. We performed sample pre-treatment, and the extraction method we used is based on Shackleton and Whitney’s extraction method [7]. After the sample preparation processes the concentrations (μg/24 hours) of the following urinary androgen, progesterone (P), and corticoid metabolites were determined by gas chromatography-mass spectrometry (GC-MS)/selected ion-monitoring (SIM): androsterone (An), etiocholanolone (Et), dehydroepiandrosterone (DHEA), 11β-hydroxyandrosterone (11-OH-An), 16-hydroxy-DHEA (16-OH-DHEA), pregnanediol (PD), pregnanetriol (PT), pregnenediol (Δ5-PD), androstenetriol (Δ5-AT), tetrahydro-11-deoxycortisol (THS), 11-keto-pregnanetriol (11-O-PT), tetrahydrocortisone (THE), tetrahydro-11-dehydrocorticosterone (THA), tetrahydrocorticosterone (THB), allo-tetrahydrocorticosterone (aTHB), tetrahydrocortisol (THF), allo-tetrahydrocortisol (aTHF), α-cortolone (α-CL), β-cortolone (β-CL), and α-cortol (α-C). All components were detected in all samples. The interventions and treatments of patient 1, the collection of urine samples, and the levels of the urinary steroid metabolites are shown in Fig. 3.

### Patient 2

**Fig. 4:**
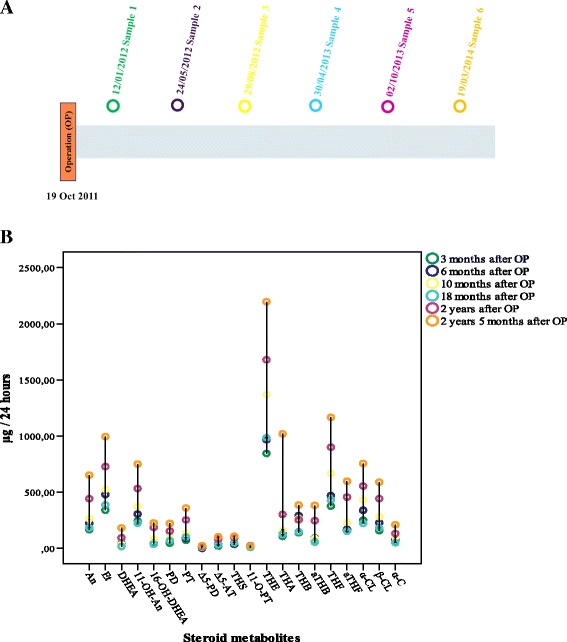
Patient 2. **a** The interventions and treatments of the patient and the collection of urine samples. **b** The levels of the urinary steroid metabolites at different time points

A 55-year-old European, postmenopausal woman was diagnosed with stage T1a ovarian GCT in October 2011. On admission, the results of her neurological examination were normal. Her physical examination was otherwise unremarkable. A CT scan depicted a large, single pelvic mass with inhomogeneous signal intensity (164×113×146 mm) and ascites. A laboratory examination did not reveal an elevated level of CA-125 (11 U/ml) tumor marker. She underwent laparotomy and the entire tumor was removed. The pathologic diagnosis was GCT. No further treatment was given. On clinical and radiological examination 11 months later, she was found to be free of the disease. After the OP, during the oncological follow-up, six 24-hour urinary samples were collected at different time points. Her urinary concentrations (μg/24 hours) of An, Et, DHEA, 11-OH-An, 16-OH-DHEA, PD, PT, Δ5-PD, Δ5-AT, THS, 11-O-PT, THE, THA, THB, aTHB, THF, aTHF, α-CL, β-CL, and α-C were determined by GC-MS/SIM. All components were detected in all samples. The interventions and treatments of patient 2, the collection of urine samples, and the levels of the urinary steroid metabolites are shown in Fig. 4.

## Discussion

GCT is hormonally active, so the qualitative and quantitative determination of steroid hormones has an important role in the follow-up and the diagnostics of this tumor. The urinary steroid profile is a feasible method, which allows us to measure several steroid groups in parallel and it is a noninvasive procedure.

In the first case, 1 month before the OP, in the first sample the urinary concentrations of An, Et, 11-OH-An, 16-OH-DHEA, Δ5-AT, PT, Δ5-PD, THE, aTHF, and α-CL were higher than the same age and same sex reference values. A laboratory examination did not reveal elevated levels of serum tumor markers and hormones. At 2 and 4 months after the OP, during the BEP chemotherapy (samples 2 and 3), the urinary levels of all metabolites were lower than the reference values. Six months after the OP (after BEP chemotherapy and metastasectomy, before CAP I chemotherapy) the urinary concentrations of An, Et, 11-OH-An, 16-OH-DHEA, Δ5-AT, PT, Δ5-PD, THE, THA, THB, THF, aTHF, α-CL and α-C were higher than the reference values (sample 4). After CAP I chemotherapy, a laboratory examination did not reveal elevated levels of serum tumor markers; however, in samples 5 to 8 (10 months, 15 months, 20 months, and 2 years 6 months after OP) the urinary concentrations of An, 11-OH-An, and Δ5-AT were higher than the reference values. In sample 9 (2 years 11 months after OP and before epirubicin + cisplatin chemotherapy) the urinary concentrations of An, Et, DHEA, 11-OH-An, 16-OH-DHEA, Δ5-AT, PT, Δ5-PD, THE, aTHF, and α-CL were found to be higher than the reference values again. In addition, a CT examination revealed that the tumor had progressed considerably. The elevated urinary hormone levels of the previous samples (samples 5 to 8) might have already indicated this progress. After epirubicin + cisplatin chemotherapy in sample 10 (4 years 1 month after OP), the urinary concentrations of six metabolites (An, 11-OH-An, Δ5-AT, PT, THE, and α-CL) were higher than the reference values; however, CT showed regression of the tumor. In sample 11 (4 years 6 months after OP and after epirubicin + cisplatin chemotherapy), the urinary concentrations of An, Et, DHEA, 11-OH-An, 16-OH-DHEA, Δ5-AT, PT, THE, THF, aTHF, α-CL, and β-CL were found to be higher than the reference values again. Under the anastrozole treatment in samples 12 and 13 (5 years 2 months and 5 years 5 months after OP), only the urinary concentration of THE was higher, the urinary concentrations of other metabolites were lower than the reference values. In all urine samples the concentrations of PD, THS, and 11-O-PT were lower than the reference values.

To summarize, before the OP the concentrations of urinary metabolites of serum androgens, pregnenolone, and 17-hydroxyprogesterone were elevated. The concentrations of the urinary metabolites of P, 21-deoxycortisol, and 11-deoxycortisol were low. These changes were found after further treatments (surgery and chemotherapies), so they referred to the presence of the recurrent GCT. The elevated levels of the urinary metabolites of cortisol and cortisone referred to the effects of the stress. The chemotherapy and the aromatase inhibitor (anastrozole) treatments amended the steroid metabolism.

In case 2 the urinary concentrations of the metabolites are presented in Fig. 4. After the OP, during a 3-year follow-up period in all urine samples (samples 1 to 6) the concentrations of 20 steroid metabolites were lower than the reference values.

The obtained urinary steroid concentrations, as patient 2 was free of the GCT after an OP, corresponded to the postmenopausal state of women without ovaries.

## Conclusions

Our results suggest that the recurrence of GCT changes urinary steroid profiles, which was indicated by the differences between the urinary steroid levels of the two patients. To confirm that the presence of a GCT can be identified based on a urinary steroid profile, we plan to carry out further multicenter clinical trials.

## Abbreviations

11-OH-An: 11β-hydroxyandrosterone
11-O-PT: 11-keto-pregnanetriol
16-OH-DHEA: 16-hydroxy-DHEA
A: Androstenediol
AFP: Alpha-fetoprotein
An: Androsterone
aTHB: Allo-tetrahydrocorticosterone
aTHF: Allo-tetrahydrocortisol
BEP: Bleomycin, etoposide, and cisplatin
CA-125: Carbohydrate antigen 125
CA-15-3: Carbohydrate antigen 15–3
CA-19-9: Carbohydrate antigen 19–9
CAP I: Cyclophosphamide and doxorubicin
CEA: Carcinoembryonic antigen
CT: Computed tomography
DHEA: Dehydroepiandrosterone
E_2_: 17β-estradiol
Et: Etiocholanolone
FSH: Follicle-stimulating hormone
GC-MS: Gas chromatography-mass spectrometry
GCT: Granulosa cell tumor
LH: Luteinizing hormone
MRI: Magnetic resonance imaging
OP: Operation
P: Progesterone
PD: Pregnanediol
PT: Pregnanetriol
SIM: Selected ion-monitoring
T: Testosterone
THA: Tetrahydro-11-dehydrocorticosterone
THB: Tetrahydrocorticosterone
THE: Tetrahydrocortisone
THF: Tetrahydrocortisol
THS: Tetrahydro-11-deoxycortisol
α-C: α-cortol
α-CL: α-cortolone
β-CL: β-cortolone
Δ5-AT: Androstenetriol
Δ5-PD: Pregnenediol
